# Prevalence of pigeon rotavirus infections: animal exhibitions as a risk factor for pigeon flocks

**DOI:** 10.1007/s00705-020-04834-w

**Published:** 2020-10-16

**Authors:** Maxi Harzer, Kristin Heenemann, Michael Sieg, Thomas Vahlenkamp, Markus Freick, Antje Rückner

**Affiliations:** 1 Center for Infectious Diseases, Faculty of Veterinary Medicine, Institute of Virology, Leipzig University, An den Tierkliniken 29, 04103 Leipzig, Germany; 2 Faculty of Agriculture/Environment/Chemistry, HTW Dresden-University of Applied Sciences, Dresden, Germany

## Abstract

A total of 289 cloacal swabs from pigeons from 29 different breeders in Germany were collected. In addition, samples from pigeons exhibited at shows were collected. The detailed health status of the pigeon flocks was recorded. Samples were analysed for the presence of the recently discovered pigeon rotavirus and pigeon circovirus. Pigeon rotavirus was found in 10.3% and pigeon circoviruses was found in 65.5% of sampled pigeon lofts. The study revealed a strong relationship between the attendance of shows and the occurrence of different clinical signs. The higher prevalence of pigeon rotavirus in exhibited animals indicates that exhibitions are a risk factor for the transmission of this pathogen.

**Electronic supplementary material:**

The online version of this article (10.1007/s00705-020-04834-w) contains supplementary material, which is available to authorized users.

## Introduction

In 2016, a disease of unknown origin emerged in Australian racing pigeons, resulting in a loss of performance and high mortality rates due to hepatitis. Further investigations revealed that the disease was associated with a novel avian rotavirus [1]. Members of the genus *Rotavirus*, family *Reoviridae*, are non-enveloped, segmented RNA viruses. Infections cause acute viral gastroenteritis in humans, as well as in other mammalian and avian species [2]. Rotaviruses are classified into nine species (A to D, F to J) based on the antigenicity of the outer capsid protein VP6 [3]. Members of the rotavirus species A, D, F and G have been found in poultry [2]. Infections are usually restricted to the gastrointestinal tract and are self-limiting, although fatalities can occur as a result of dehydration, especially in younger animals. In contrast, the newly described pigeon‐associated clade of rotavirus A (RVA) has been shown to cause systemic infections [4], resulting in clinical signs such as apathy, fluffy plumage, inappetence, emesis, stowed crop, emaciation, and diarrhoea [1, 4, 5]. So far, viruses of this clade have only been detected in pigeons. Recently, an infection study fulfilled Henle-Koch's postulates and confirmed pigeon RVA genotype G18P[17] to be primary cause of young pigeon disease syndrome (YPDS)-like diseases in domestic pigeons [5]. Furthermore, it is supposed that the pigeon circovirus (PiCV), a member of the genus *Circovirus*, family *Circoviridae*, contributes to outbreaks of YPDS. The pathogenesis of PiCV-associated disease has not yet been conclusively clarified. Lymphoid depletion and lymph cellular necrosis have been observed in the spleen and bursa of Fabricius. The findings obtained so far suggest an immunosuppressive property of this viral infection, which may favour secondary infections. This possibility was supported by the observation that YPDS can be associated with high morbidity and mortality rates under different circumstances with varying disease signs [6–8]. Breeders have often reported YPDS outbreaks in pigeon lofts after participation in shows. We therefore investigated the presence of pigeon RVA and PiCV in animals exhibited at shows and compared these results to the observed clinical symptoms in the affected animals.

## Materials and methods

Cloacal swabs of pigeons from different lofts located in Germany were collected and investigated for the presence of pigeon RVA and PiCV using RT-PCR and PCR, respectively. In total, 289 samples were collected between December 2017 and February 2018, up to 5 months after the appearance of different clinical symptoms. The health status of the pigeon flocks was recorded using a questionnaire. In addition, mortality and morbidity rates, medical treatments (e.g., antibiotic therapy), and information about the date when symptoms first occurred were requested from the owners. By this approach, the following samples were included into the study: In total, 29 different flocks comprising 15-250 individual squeakers were included by sampling 10 randomly selected animals per flock, except for one flock, from which nine swabs were sent. Swabs from five stocks were sent in 2017 and from 24 stocks in 2018. In addition to these pigeon flocks, we obtained cloacal swabs from 10 pigeons that were left at the end of a poultry exhibition in Saxony in 2017. Liver samples were taken from 10 pigeons that died at a poultry show in December 2018. Liver and cloacal swabs were taken from five pigeons that died at a poultry show in December 2019. Currently, autogenous RVA vaccines and Colvac RP (Pharmagal s.r.o.), a commercial inactivated RVA vaccine for pigeons licensed 2019 in the Czech Republic, are in use in Germany. The vaccine contains RVA of the genotype G18P[17] and pigeon paramyxovirus 1. To compare the licensed vaccine with the currently circulating RVA strains in pigeons, we sequenced the RVA isolates and performed phylogenetic analysis to examine their relationship to previously described pigeon RVA and PiCV isolates.

Swabs were suspended in Dulbecco's phosphate-buffered saline (DPBS, Thermo Fisher Scientific) and stored at -80 °C until analysis. Livers were stored in the same way. Subsequently, RNA and DNA were extracted using an RNeasy Mini Kit and a DNeasy Blood & Tissue Kit according to the manufacturer’s instructions (QIAGEN). The RVA genome was amplified by RT-PCR with the viral genome-segment-6-specific primers VP6AvRVA s (5’-CARCCWGCKCAYGATAATGTNTGTGG-3´) and VP6AvRVAas (5’-GTCCARTTCATWCCHGCWGGAAATACTGG-3´), which were designed on the basis of already existing genetic information of the VP6 genes of avian RVA strains. The specificity of the RT-PCR was confirmed by testing different control RVA strains of avian and mammalian origin (RVA strains B223, OSU, SA11, turkey field strains, and pigeon RVA G18[17]). Sensitivity was quantified by using a plasmid control. For this purpose, a primer-flanked PCR product was cloned into the plasmid pJet1.2 in accordance with the instructions of the manufacture of the CloneJET™ PCR cloning kit (Thermo Fisher Scientific). A detection limit of 200 copies per reaction was determined. For sample analysis, RNA preparations from five pooled swabs were subjected to cDNA synthesis using Superscript III reverse transcriptase (Thermo Fisher Scientific) according to the manufacturer’s instructions, applying the following thermal profile: 95 °C for 5 min, 50 °C for 60 min, and a final step at 85 °C for 5 min. Superscript III reverse transcriptase was added after the initial denaturation step. For amplification of a portion of the VP6 gene, 4 µl of cDNA was mixed with 5 µl of 10x Dream Taq Buffer including 20 mM MgCl_2_ (Thermo Fisher Scientific), 1 µl of dNTPs (final concentration, 0.2 µM), 1 µl of each primer (final concentration, 0.2 µM) and 0.25 µl of Dream Taq Polymerase (final concentration, 0.025 U/µl) (Thermo Fisher Scientific). The PCR protocol started with the activation of the polymerase for 3 min at 95 °C, followed by 35 cycles of denaturation for 30 s at 95 °C, annealing for 30 s at 50 °C, and elongation for 30 s at 72 °C. The reaction ended with a final elongation step for 5 min at 72 °C. If pooled samples gave a positive result, they were analysed individually as described above. Subsequently, the segments encoding viral proteins 4 and 7 of the RVA-positive samples were amplified. The following primers were designed on the basis of already existing genetic (information for the pigeon RVA strains: aRVA VP4 s (5’-GGCTATAAAATGGCTTCTC-3´) aRVA VP4 as (5’-GTCACATCCTCATAGACA-3´) aRVA VP7 s (5’-TTCTCACCGCGATTAG-3´) and aRVA VP7 as (5’- TATACCCTCAAAAAGTATGC-3´). For cDNA synthesis and PCR, the same reagents were used as in the protocol described for VP6. The thermal profile was adapted to the primers and the size of the amplified gene segment. The annealing temperature in the step of DNA amplification was 53 °C, and the elongation time was extended to 2 min or 1 min for VP4 and VP7 gene segment amplification, respectively. Pooled samples were screened for the presence of PiCV applying a consensus nested PCR as described previously [9]. In addition, cloacal swabs from pigeons that were abandoned at an exhibition in Saxony were tested for the presence of avian orthoreovirus, psittacid herpesvirus 1, and pigeon aviadenoviruses A and B as described previously [10–12]. All PCR products were visualized by agarose gel electrophoresis (Tris-acetate-EDTA buffer, pH 8.3, containing 0.2 µg of ethidium bromide per ml). Positive pigeon RVA PCR reaction products were purified using a GeneJET PCR Purification Kit (Thermo Fisher Scientific) and subjected to sequencing by the Sanger dideoxy termination method (Microsynth Seqlab GmbH). From among the pigeon-RVA-positive pigeons, PiCV-positive pools were analysed individually, and the PCR product were purified and sequenced. The resulting nucleotide sequences were used to carry out a database search at the NCBI (National Center for Biotechnology Information) website, using the Basic Local Alignment Search Tool (BLAST). The software MEGA X was used for phylogenetic analysis and construction of phylogenetic trees.

## Results

**Fig. 1:**
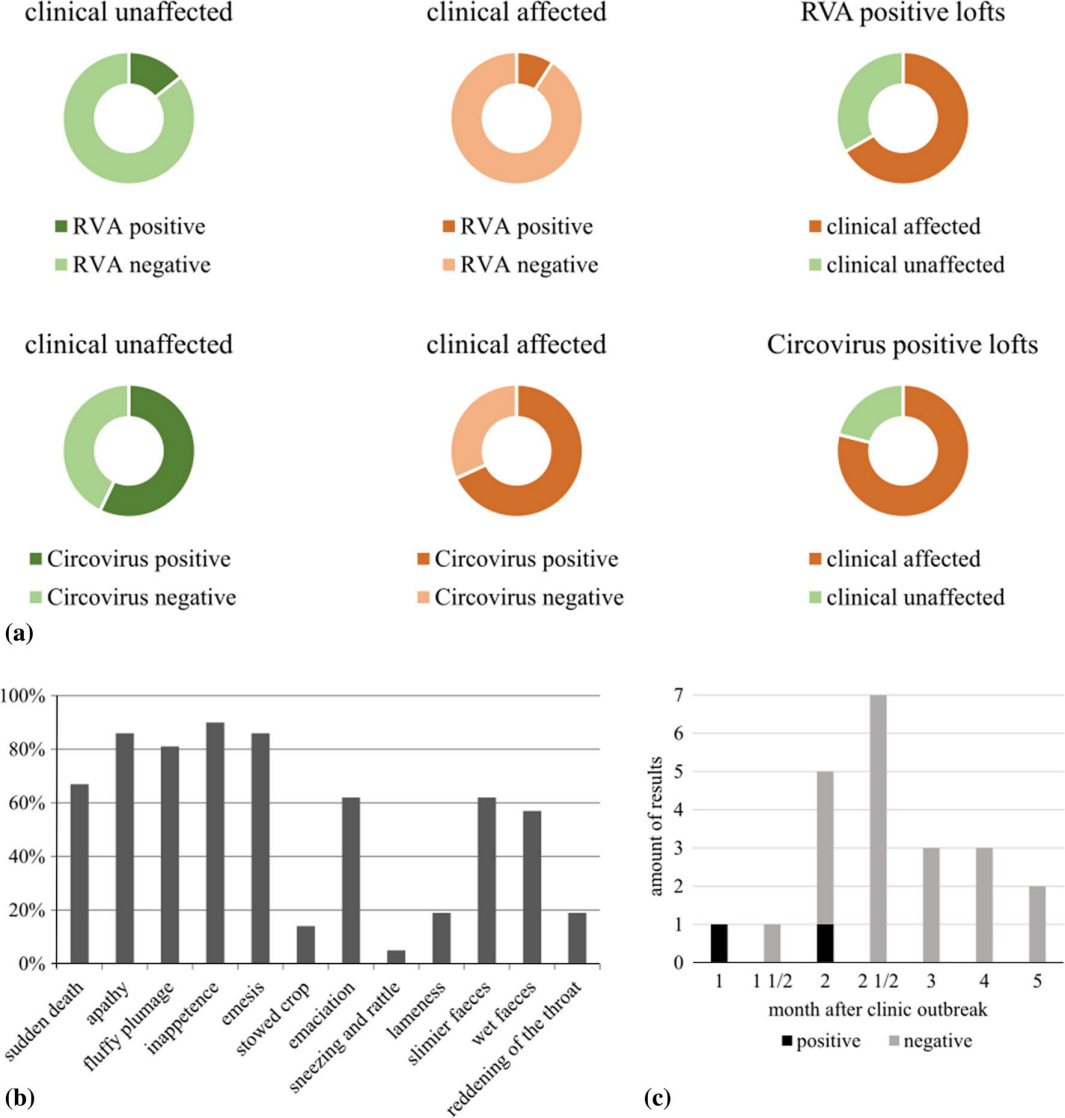
Occurrence of clinical signs and virus detection in different lofts. (a) Distribution of RVA or PiCV results among affected or unaffected lofts and clinical observations within positive tested groups. (b) Clinical symptoms, reported in the questionnaire. (c) Correlation between RVA results and the time between the clinical outbreak and the sample collection date

Out of 29 pigeon lofts analysed, 22 (75.9%) were reported by pigeon breeders to have experienced clinical signs immediately or a few days after participation in breeder shows, while the remaining 25% of the participants did not report any signs. As shown in Fig. 1, pigeons in more than three-quarters of the lofts have clinical signs within 1 to 4 days after visiting a show, although the animals were previously given a health certificate by the attending veterinarian and no signs of disease were evident prior to the exhibition.

In all cases, the observed signs were reported to have occurred in the time period of September to December 2017. The mortality ranged from 0% to 75%, whereas the morbidity ranged from 8% to 100%. Most affected squeakers were between the age of 3 weeks and 8 months.

Three out of 29 flocks (10.3%) tested positive for pigeon RVA, whereas negative results were obtained in both clinical and nonclinical stocks at the time of sampling. Six of seven (85.7%) inconspicuous lofts tested negative for RVA. Affected lofts had a detection rate of 9.1% (2/22) for pigeon RVA. Within the pigeon-RVA-positive group, 66.6% of the animals showed clinical signs at the time point of sampling.

Overall, 19 out of 29 flocks (65.5%) tested positive for PiCV. In reference to the whole study group, 15 of 22 clinically affected lofts (68.1%) and four of seven clinically unaffected lofts (55%) were positive for PiCV. Within the PiCV-positive group, 78.9% showed clinical signs (Fig. 1).

The first pigeon-RVA-positive flock identified, composed of 30 Saxon croppers and Silesian pouters, showed clinical signs such as inappetence, emesis, and emaciation. Analysis of individual swabs resulted in a detection rate of 50%. All animals were affected by clinical symptoms, and three pigeons died during the observation period. Furthermore, PiCV was detected in this flock. Clinical symptoms occurred immediately after attendance of the exhibition, and swabs were sent within 1 month to our laboratory while the symptoms persisted. The owner of the second flock, composed of 29 Thuringian croppers, did not report any disease or symptoms in his loft, while 20% of the single swabs tested positive for RVA. Swabs were sent 2 month after the attendance of an exhibition. The third pigeon-RVA-positive flock, composed of 54 rhinestone pigeons, lost 24 of 33 squeakers, pursuant to a mortality rate of 75%, even though just 30% of the individually tested swabs were positive for RVA. All animals were diseased and exhibited apathy, inappetence, emesis, altered faeces, lameness, and emaciation. No PiCV was detected, but mild infestation with *Salmonella*, coliform bacteria, and coccidia was reported in the questionnaire. Swabs were sent to our laboratory 2 months after the first clinical signs, which lasted over 2 months. The owner reported participation in an exhibition with his pigeons two days before the clinical signs occurred.

The time period between first outbreak of the disease and sampling ranged from a few weeks to four months. With an increasing time span between the occurrence of clinical signs and the date of sampling, fewer samples tested positive for RVA (Fig. 1). The owners of the three positive flocks described above sent swab samples within 2 months after visiting an exhibition and the onset of clinical signs. In total, 12 lofts sent swabs more than 2 months after the first clinical symptoms appeared. There were no acute clinical signs at the time of signs collection.

Five out of ten birds that were abandoned at a poultry exhibition in 2017 tested positive for RVA, and four were found to be positive for PiCV*.* Additional testing for avian orthoreovirus, psittacid herpesvirus 1, and pigeon aviadenovirus A and B gave negative results. In 2018, nine out of 10 livers taken from pigeons that died at a poultry exhibition in Saxony tested positive for pigeon RVA and PiCV (90%). In 2019, livers and cloacal swabs were taken from five pigeons that died at a poultry exhibition in Saxony. Four tested positive for pigeon RVA (80%) and two for PiCV (20%). One sampled pigeon had a negative result for RVA in the cloacal swab and a positive result for RVA in the liver (Table 1). This corresponds to RVA detection rates of 50%, 90%, and 80% from 2017 to 2019. There were no macroscopic lesions in any of the organ samples. Histological examinations were not performed.

**Table 1:** Results obtained with samples from pigeons at exhibitions in 2017, 2018, and 2019. The table shows file information on the number of samples analysed per year and the RVA and PiCV (RT)-PCR results. The genotypes of RVA isolates are listed. Pigeon “1” from the year 2019 was negative for RVA in the cloacal swab but positive in the liver. All other pigeons showed a positive or negative result in both samples. In addition, some samples were positive for PiCV by PCR

Pigeon number	Year of exhibition	Material	RVA result	Genotyping result	PiCV result
1	2017	Cloacal swab	Negative		Negative
2	2017	Cloacal swab	Negative		Negative
3	2017	Cloacal swab	Negative		**Positive**
4	2017	Cloacal swab	**Positive**	G18[P17]	Negative
5	2017	Cloacal swab	**Positive**	G18[P17]	Negative
6	2017	Cloacal swab	**Positive**	G18[P17]	Negative
7	2017	Cloacal swab	Negative		**Positive**
8	2017	Cloacal swab	Negative		Negative
9	2017	Cloacal swab	**Positive**	G18[P17]	**Positive**
10	2017	Cloacal swab	**Positive**	G18	**Positive**
1	2018	Liver	**Positive**	G18[P17]	**Positive**
2	2018	Liver	**Positive**	G18[P17]	**Positive**
3	2018	Liver	**Positive**	G18[P17]	**Positive**
4	2018	Liver	**Positive**	G18[P17]	**Positive**
5	2018	Liver	**Positive**	-	Negative
6	2018	Liver	**Positive**	G18[P17]	**Positive**
7	2018	Liver	**Positive**	G18[P17]	**Positive**
8	2018	Liver	Negative		**Positive**
9	2018	Liver	**Positive**	G18[P17]	**Positive**
10	2018	Liver	**Positive**	G18[P17]	**Positive**
1	2019	Liver	**Positive**	G18	Negative
1	2019	Cloacal swab	Negative		Negative
2	2019	liver	**Positive**	G18[P17]	Negative
2	2019	Cloacal swab	**Positive**	-	**Positive**
3	2019	liver	Negative		Negative
3	2019	Cloacal swab	Negative		Negative
4	2019	liver	**Positive**	G18	Negative
4	2019	Cloacal swab	**Positive**	-	Negative
5	2019	liver	**Positive**	G18[P17]	**Positive**
5	2019	Cloacal swab	**Positive**	G18[P17]	Negative

**Fig. 2:**
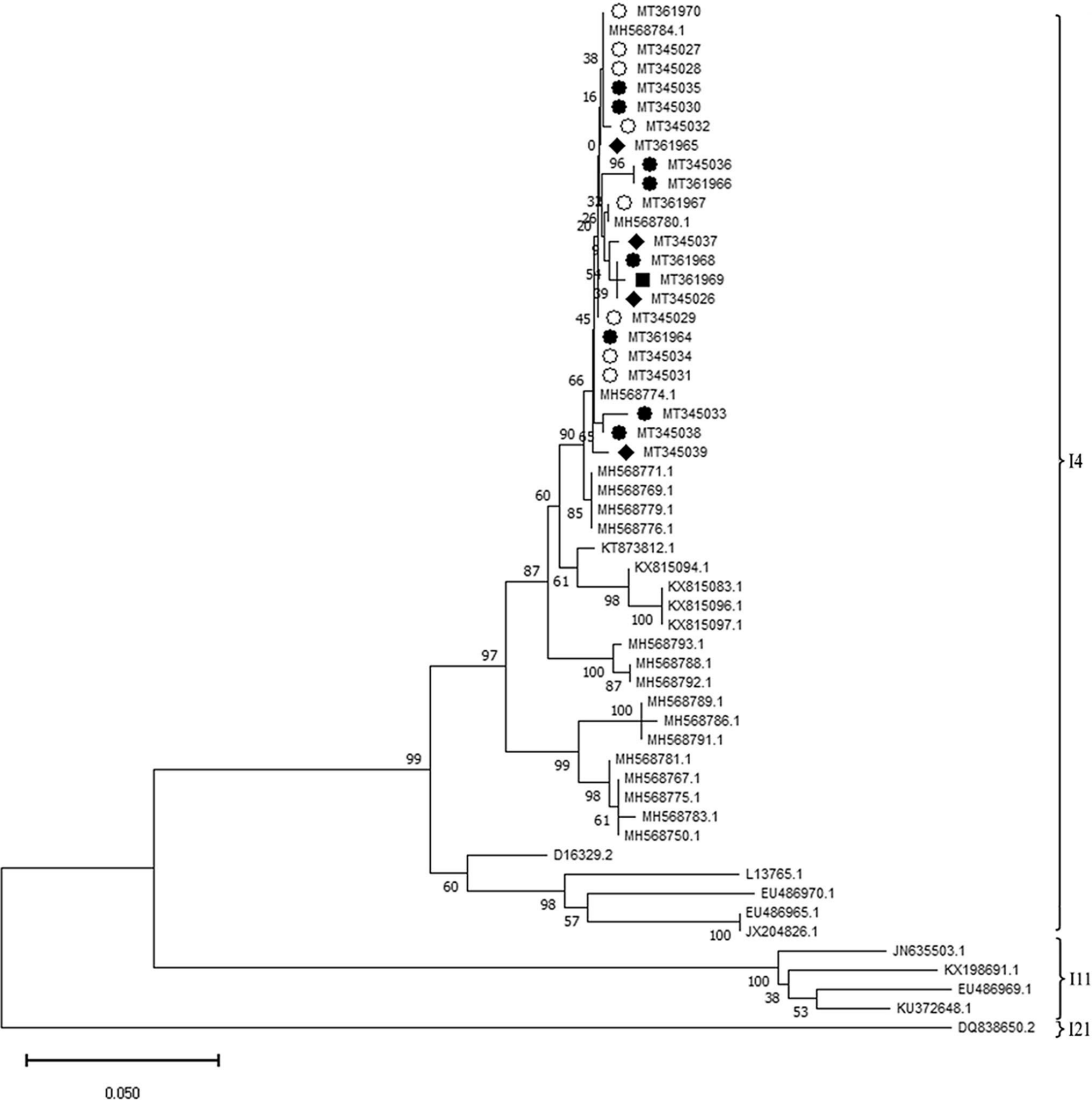
Phylogenetic analysis based on VP6 sequences. Partial VP6 sequences (519 nt) were analysed in comparison to sequences of the typical avian RVA genotypes I4, I11, and I21 obtained from GenBank as described [4]. Genotype assignments are based on an 81% nucleotide sequence identity cutoff [3]. The tree was built using the neighbour‐joining algorithm and the Jukes‐Cantor distance model in MEGA X. Values at branches represent percent branch support for 1,000 bootstrap replicates. Pigeon RVA isolated from pigeon samples from lofts, ○; samples from an exhibition in 2017, ■; samples from an exhibition in 2018, ●; samples from an exhibition in 2019, ♦

**Fig. 3:**
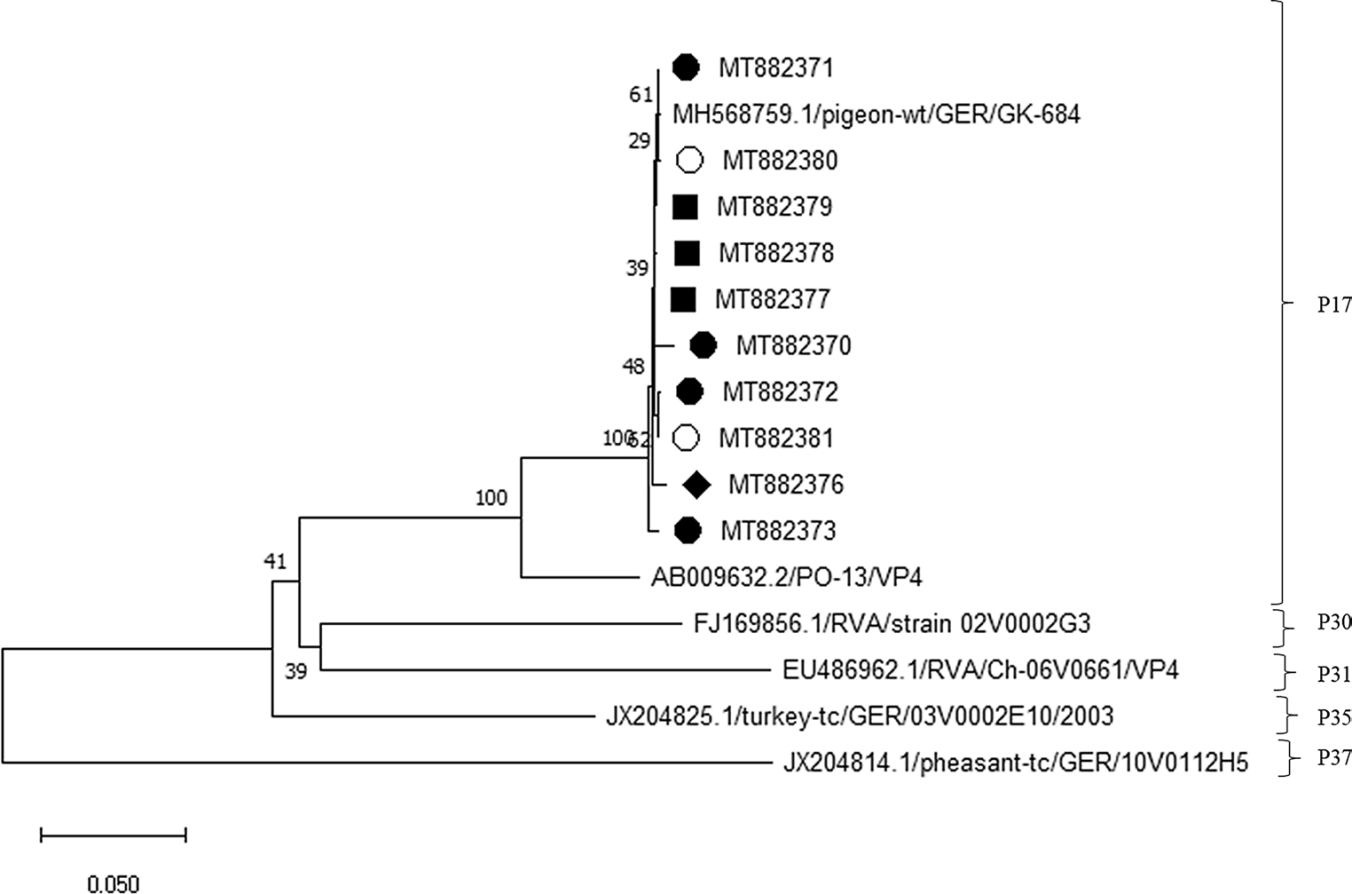
Phylogenetic analysis based on VP4 sequences. Partial VP4 sequences (660 nt) were analysed in comparison to sequences of the avian RVA genotypes P[17], P[30], P[31], P[35], and P[37] obtained from GenBank. Genotype assignments are based on a previously established cutoff value [13]. The tree was built using the neighbour‐joining algorithm and the Jukes‐Cantor distance model in MEGA X. Values at branches represent percent branch support for 1,000 bootstrap replicates. Pigeon RVA isolated from pigeon samples from lofts, ○; samples from an exhibition in 2017, ■; samples from an exhibition in 2018, ●; samples from an exhibition in 2019, ♦

**Fig. 4:**
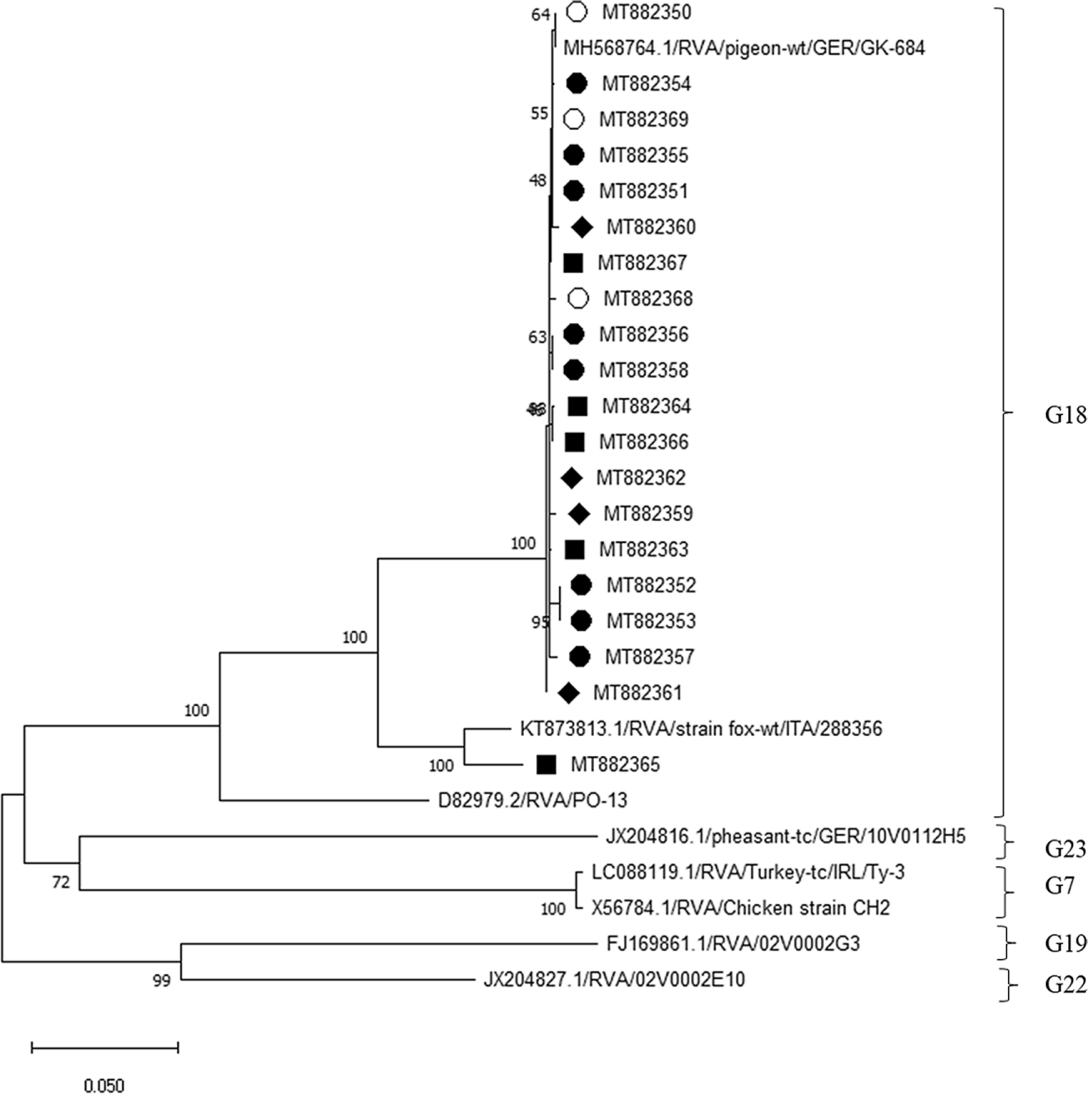
Phylogenetic analysis based on VP7 sequences. Partial VP7 sequences (845 nt) were analysed in comparison to sequences of the avian RVA genotypes G7, G18, G19, G22 and G23 obtained from GenBank. Genotype assignments are based on a previously established cutoff value [13]. The tree was built using the neighbour‐joining algorithm and the Jukes‐Cantor distance model in MEGA X. Values at branches represent percent branch support for 1,000 bootstrap replicates. Pigeon RVA isolated from pigeon samples from lofts, ○; samples from an exhibition in 2017, ■; samples from an exhibition in 2018, ●; samples from an exhibition in 2019, ♦

The VP6 genes of these RVA strains showed a high level of nucleotide sequence identity to each other, ranging between 99% and 100%. The analysis of partial VP6 gene sequences (519 nt) from the pigeon RVA detected in this study revealed that these isolates clustered with genotype I4 [4] (Fig. 2). Further genetic analysis of the viral proteins 4 and 7 showed that the detected pigeon RVA virus belonged to a clade that had already been described in pigeons (Figs. 3 and 4). RVA within this clade are of genotype G18P[17] and are grouped into one of the avian subgroups of RVA [1].

## Discussion

Most of the lofts included in this study reported clinical signs like those associated with pigeon RVA infection [4] and YPDS [8]. There was a conspicuous correlation with prior attendance of shows. The prevalence of pigeon RVA among clinically affected pigeon lofts turned out to be much lower than in other studies [4, 5]. Most of the analysed samples from the pigeon lofts were sent months after the first symptoms occurred. It has been observed that pigeon RVA can be shed for three months after infection [4]. It is therefore conceivable that RVA may have been circulating in the loft at the time when clinical signs were apparent but was no longer excreted at the time of sample collection. PiCV was spread almost equally among the affected and unaffected groups and therefore cannot be identified as a single trigger for the observed clinical signs. Genetic analysis of the PiCV isolates from this study (accession numbers MT913339-MT913350) revealed that they clustered with various other PiCV isolates (Supplemental Material S1). There was no unique PiCV strain that was associated with shows, and there was also no PiCV strain that was especially associated with pigeon RVA infections. The identification of virus in the blood may be an efficient way to differentiate between infected and uninfected animals, and this could sharpen the clinical significance [14]. In general, PiCV is believed to cause immunosuppression and may facilitate secondary infections [8]. As seen in the rhinestone pigeon loft, viral and bacterial coinfections, especially with RVA involvement, may aggravate the clinical picture of other infections. This has been shown for other avian and mammalian species [15]. Confirmation of this hypothesis requires further investigation, as an experimental RVA infection experiment did not show any losses [5].

Because first clinical signs in nearly all flocks occurred during September and December after participation in shows, we suppose that participation in such shows is associated with a significantly increased risk of infection with different pathogens. The detected prevalence of 50%, 80% or 90% for RVA among the sampled pigeons at exhibitions provides a strong basis for the spread of these pathogens. In addition, the pigeon RVA isolates that were detected showed a high degree of genetic similarity to each other, suggesting that circulation of these viruses occurs in the pigeon population. The genetic information in the VP6, VP4 and VP7 gene segments did not mutate significantly during this period, and the same strains are still circulating. The strains in this study showed the highest similarity to the main strains circulating in 2017 [4]. From rotaviruses, it is known that a low infectious dose is sufficient to cause an infection and a disease outbreak [16]. Long-lasting excretion with high viral loads, especially in the acute phase, has been reported [4]. As a consequence, a few infected birds are sufficient for the infection of many other pigeons at a show, either directly or indirectly, since rotaviruses are very stable in the environment and remain infectious for months. The higher detection rate in samples from pigeons at exhibitions suggests that there is an accumulation and spread within the pigeon population during longer exhibitions. As seen in the sample taken from a pigeon at the exhibition in 2019, a negative result for RVA in cloacal swabs is not conclusive evidence that the pigeon is free of infection, as the virus was detected in the liver. It remains open whether the virus remain longer in the liver during infection or if it is shed discontinuously in the faeces. A single negative test for RVA does not guarantee freedom from this pathogen. PiCV is very stable in the environment and is shed via feather dust [17], so spreading at shows is possible. In contrast to the RVA results, similar detection rates of PiCV were seen in the pigeons of the lofts as well as in the birds sampled at shows. In conclusion, our investigation revealed that the attendance of exhibitions poses a risk for the spread of pigeon RVA.

Further investigations of the prevalence and incidence of RVA infections in pigeon dovecots should include monitoring over a longer period of time. In addition, samples should be collected prior to and after visits to shows to assess the risk of infection at such events in further detail. Special attention should also be given to non-living vectors such as water, food, bedding, and presentation tables. Pre-emptive rotavirus vaccination shows strong protection from clinical signs in humans [18], and as printed in the instruction leaflet of the first licensed pigeon RVA vaccine, the vaccine is able to reduce mortality and the frequency and severity of clinical signs caused by pigeon RVA infection. Sequencing of the vaccine virus revealed a high degree of similarity to circulating pigeon RVA strains.

## Electronic supplementary material

Below is the link to the electronic supplementary material.

Supplementary material 1 (PNG 34 kb)
