# Supplementary figures and images for: Prevalence of pigeon rotavirus infections: animal exhibitions as a risk factor for pigeon flocks
Source: Arch Virol. 2020 Oct 16;166(1):65–72. doi: 10.1007/s00705-020-04834-w (PMC7815556; doi:10.1007/s00705-020-04834-w)

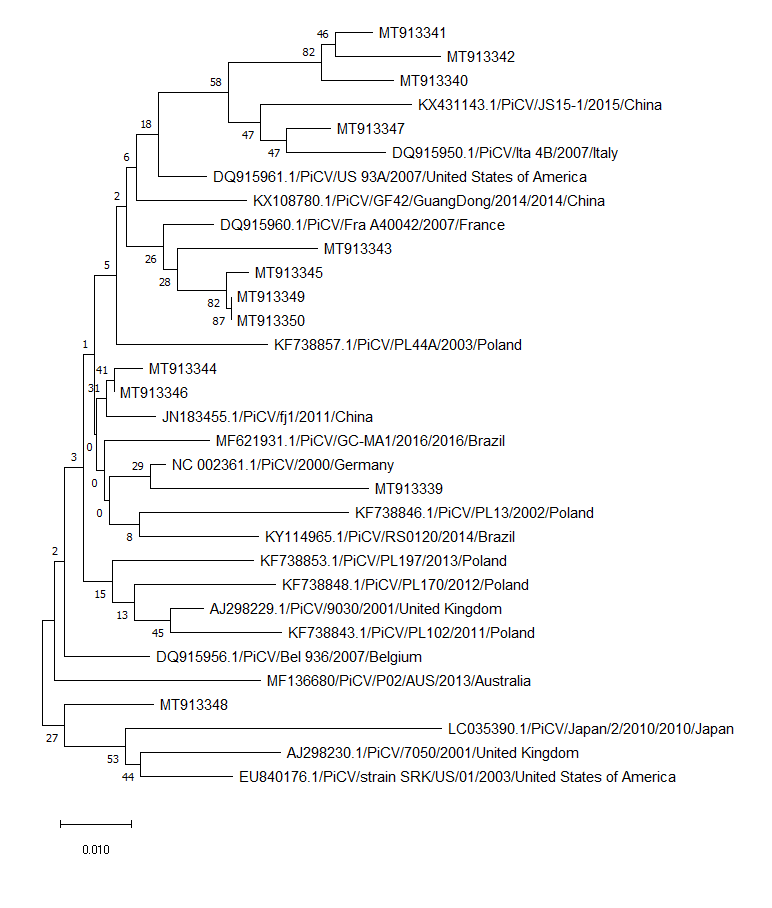

Supplement: Supplementary file 1 — Supplementary material 1 (PNG 34 kb) [file 705_2020_4834_MOESM1_ESM.png]
